# The value of histopathologic examination and Xpert (MTB/RIF) assay in diagnosis of cervical lymph node tuberculosis after coarse needle biopsy guided by CEUS: a retrospective analysis of 612 cases

**DOI:** 10.1007/s10096-024-04913-9

**Published:** 2024-08-01

**Authors:** Wenzhi Zhang, Jianping Xu, Lin Zhang, Tu Ni

**Affiliations:** Department of Ultrasonography, Hangzhou Red Cross Hospital (Integrated Chinese and Western Hospital of Zhejiang Province), Hangzhou, Zhejiang China

**Keywords:** Cervical, Lymph node, Tuberculosis, Coarse needle biopsy, CEUS

## Abstract

**Objective:**

To investigate the value of histopathological examination (HPE) and Xpert Mycobacterium tuberculosis bacilli/rifampicin (MTB/RIF) assay in diagnosis of cervical lymph node tuberculosis (LN TB) after coarse needle biopsy (CNB).

**Methods:**

We retrospectively analyzed 612 samples obtained from October 2017 to August 2023 from patients suspected cervical LN TB with surgically pathological, microbial culture confirmed, and clinically confirmed cervical lymph node enlargement who received ultrasound-guided CNB assisted by contrast-enhanced ultrasound (CEUS) at our hospital. All specimens were assessed by HPE and the Xpert (MTB/RIF) assay. We analyzed the results to determine the diagnostic value of HPE and Xpert (MTB/RIF) assay in samples taken after CEUS-assisted CNB of LN TB, and to evaluate the safety of CNB.

**Results:**

Based on the comprehensive reference standard established in this study, 532 of 612 patients were diagnosed with cervical LN TB, of which 476 were CNB positive cases, the positive rate of diagnosis was 89.5%。The sensitivity, specificity, positive predictive value, negative and predictive value of HPE were 80.4%, 91.2%, 98.4%, 41.2% respectively, while those of the Xpert MTB/RIF assay were 75.7%, 98.7%, 99.7%, 38.0% respectively. No postoperative complications were noted, and the Clavien–Dindo grade was 2.

**Conclusion:**

CEUS-assisted CNB has high diagnostic value and is safe for cervical LN TB. The sensitivity of HPE is slightly higher than that of Xpert (MTB/RIF) assay, and the specificity of Xpert (MTB/RIF) assay is higher than that of HPE, so Xpert (MTB/RIF) assay can correct the cervical lymph node tuberculosis with negative HPE.

## Introduction

Lymph node tuberculosis (LN TB) is the most prevalent form of extrapulmonary tuberculosis (EPTB), The World Health Organization’s(WHO ) Global Tuberculosis Report 2023 estimates 10.6 million new cases of TB worldwide in 2022 [1]。In China, the estimated number of new TB cases in 2022 is 748,000, with an incidence rate of 52 per 100,000, accounting for 7.1% of global TB cases [2–4].

Owing to the spread of drug-resistant tuberculosis strains, the incidence of the disease has been increasing each year, and the main clinical manifestation of cervical LN TB is cervical lymph node enlargement. The presence of drug-resistant strains can complicate the evaluation of treatment effectiveness. Thus, for patients with newly diagnosed or relapsed cervical LN TB, it is crucial to rule out the presence of drug-resistant strains. Routine pathological examination and Xpert *Mycobacterium tuberculosis* bacilli/rifampicin (MTB/RIF) assay of specimens obtained through coarse needle biopsy (CNB) can meet these clinical needs. In this study, we used contrast-enhanced ultrasound (CEUS)-assisted CNB as a diagnostic tool for cervical LN TB and evaluated the effectiveness and safety of this technique. The purpose of this study was to summarize and analyze the value of HPE and Xpert (MTB/RIF) assay of CNB specimens guided by CEUS in the diagnosis of cervical LN TB.

## Materials and methods

### Study design

We included 612 patients suspected of cervical LN TB from October 2017 to August 2023 who were later confirmed by surgical pathology, microbial culture, and clinical diagnosis. They had a history of PPT(+), repeated low fever, tuberculosis in other places, and lymph node suppuration. Their clinical manifestations included neck mass, neck discomfort, mild tenderness and neck skin swelling, and ulcer; those patients who had not responded to previous antibiotic treatment.

**Fig. 1:**
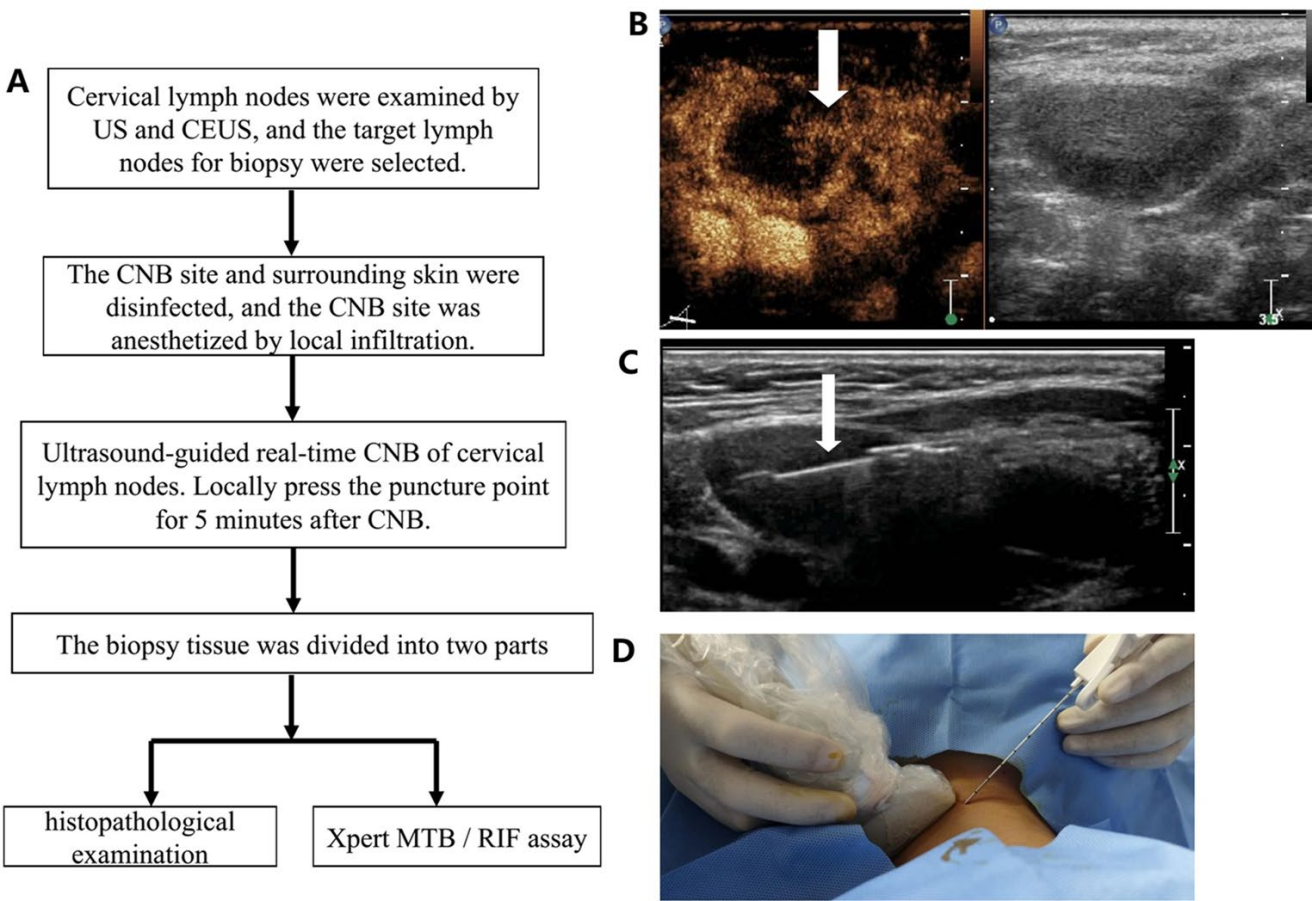
**A** is the flow chart; **B** is CEUS of cervical LN TB, lymph node showed heterogeneous enhancement, arrow shows enhancement area. **C** is US-CNB for cervical lymph node, the enhanced area was sampled, and the arrow shows the biopsy needle groove. **D** is the US-CNB process

All patients underwent CEUS and CEUS-assisted US-CNB for suspected cervical LN TB (Fig. 1). The criteria for US-CNB in patients were as follows: (1) suspected cervical tuberculosis; (2) lymph node with longitudinal diameter > 1 cm, transverse diameter > 0.5 cm, and aspect ratio < 2; (3) no history of antituberculosis therapy, no coagulation dysfunction, normal platelet range, and no serious cardiovascular and cerebrovascular diseases; (4) mental capacity to cooperate with the US-CNB procedure; (5) age > 18 years. Tissue samples were collected using CEUS-assisted US-CNB. All tissue samples were examined by histopathological examination (HPE) and the Xpert MTB/RIF assay. In this study, positive CNB cases were those that tested positive by HPE, Xpert MTB/RIF assay, or both. For patients with abscesses in cervical lymph nodes, we collected as many abscess samples as possible using a syringe and performed Xpert MTB/RIF assay and MTB culture to assist in diagnosis. This study was approved by the Human Research Ethics Committee of Hangzhou Red Cross Hospital (2022-YS-151). All methods were carried out in accordance with relevant guidelines and regulations or declaration of Helsinki.

We selected a comprehensive reference standard (CRS) to determine the diagnostic accuracy of CEUS-assisted US-CNB for cervical LN TB. The CRS included three components: positive MTB culture, pathological diagnosis of postoperative cervical lymph node CNB specimens (pathological diagnosis of surgically excised specimens), and effective antituberculosis therapy(Lymph node size was reduced during anti-tuberculosis treatment). According to the content of CRS, those with more than one positive item were considered to have LN TB, while those with all negative items were excluded.

### US and CEUS examination

We used a Philips ultrasonic diagnostic instrument (iU22, Philips Healthcare, Bothell) equipped with a high-frequency linear array probe (L12-5, frequency 5–12 MHz; L9-3, frequency 3–9 MHz) and low-frequency convex array probe (C5-1, frequency 1–5 MHz) for imaging. Bilateral cervical lymph nodes were examined using routine ultrasound. Lymph nodes with abnormal structure and large volume on the affected side were typically chosen as targets for CNB.

CEUS examination was performed using low mechanical index (0.06) pulse reverse harmonic imaging and the sulfur hexafluoride microbubble ultrasonic contrast agent SonoVue (Milan, Italy, Bracco SpA). We injected 2.4 ml SonoVue into the elbow vein, followed by an injection of 5 ml of saline, and observed the enhancement of the lesion dynamically for 2 min. All images were stored on the instrument’s hard drive for subsequent CEUS-assisted CNB.

### Diagnostic sample collection and processing

**Fig. 2:**
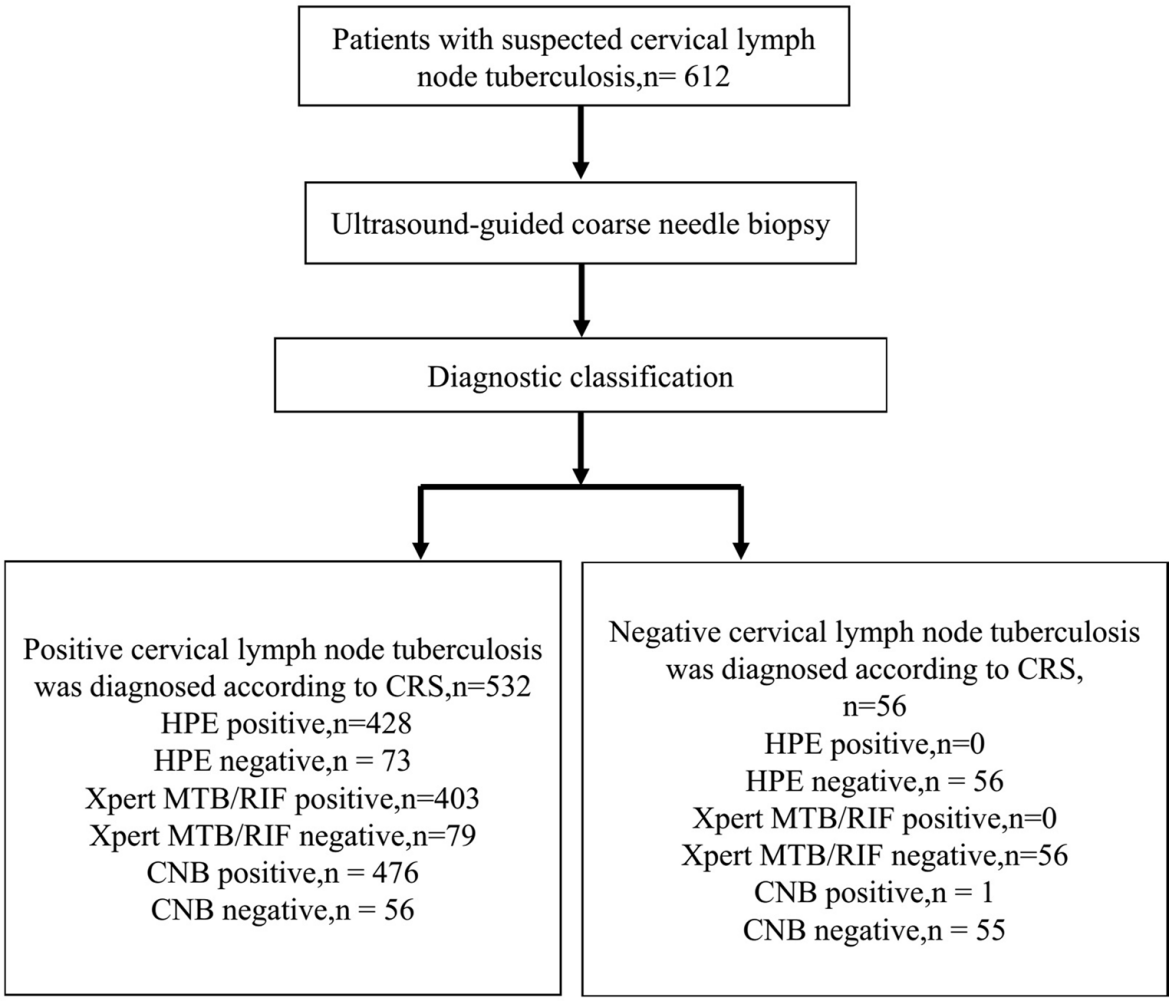
Test results of CNB in patients with suspected cervical LN TB. CNB positive: the positive result of HPE or Xpert MTB/RIF assay or presence of both findings; CNB negative: the negative result of HPE and Xpert MTB/RIF assay. CNB = coarse needle biopsy; CRS = composite reference standard; HPE = histopathology examination; MTB = Mycobacterium tuberculosis bacilli; RIF = rifampicin

We obtained tissue samples from diseased lymph nodes (enhanced areas within lymph nodes were selected as much as possible to avoid biopsy of necrotic tissue) under the guidance of CEUS and ultrasound real-time guidance using a semiautomatic biopsy gun (18G, TSK, Japan) (Fig. 1). The biopsy length was approximately 1.0–2.0 cm, and samples were collected twice in different directions. The puncture site was anesthetized prior to biopsy, and pressure was applied to the puncture site for 10 min after biopsy to prevent bleeding (Fig. 1). Biopsy tissue specimens were typically divided into two parts, one for HPE and the other for Xpert MTB/RIF detection (Fig. 2). For necrotic lymph nodes shown by CEUS, use a syringe to collect as much pus as possible and send it for TB culture.

### HPE

Tissue samples were fixed in 10% formalin, embedded in paraffin, and cut into 5-µm-thick sections, which were stained with hematoxylin–eosin (HE) or Ziehl–Neelsen stain and evaluated by HPE. Positive HPE results in Presence of chronic granulomatous inflammation with caseous necrosis; acid-fast bacteria (AFB) on Ziehl–Neelsen staining, or both [5, 6]. (1) Positive.

acid-fast staining of smear or pathological section; (2) Presence of.

chronic granulomatous inflammation with caseous necrosis;

### Xpert MTB/RIF assay

A tissue sample processing reagent (comes with the Xpert MTB/RIF reaction kit) was added to the tissue sample, which was then ground until a suspension was formed. The suspension was transferred to a sterile conical tube, shaken 20 times, and incubated at room temperature for 10 min. The conical tube was shaken 20 more times and incubated for an additional 5 min. Finally, 2 ml of the treated sample was transferred to the Xpert MTB/RIF reaction kit (Cepheid, Sunnyvale, California) using a new pipette. The reaction kit can detect TB automatically.

### Perioperative prognostic evaluation of CNB specimens

Perioperative outcomes included blood loss, duration of operation, and length of hospital stay. In addition, we recorded complications within 7 days after surgery based on patient charts using the Clavien–Dindo classification method [7].

### Data processing and statistical analysis

We compared the positive rate of diagnosis of cervical LN TB using HPE and the Xpert MTB/RIF assay. All data were analyzed using SPSS ver. 23.0 statistical software (USA, IBM). Numerical data and differences between HPE and Xpert MTB/RIF assay were analyzed using χ^2^ and Fisher’s exact tests. A *P* value of < 0.05 was considered statistically significant.

## Results

### Patients

A total of 612 patients with suspected cervical lymph node TB who underwent CEUS-assisted US-CNB were included in this study. The mean age of the patients was 31.54 ± 10.21 years (range: 19–68 years), and 340 (55.6%), 201 (32.8%), and 71 (11.6%) patients were aged 20–40, 40–60, and > 60 years, respectively. According to the CRS established in this study, 532 patients were diagnosed with cervical LN TB, 34 with necrotizing lymphadenitis, 18 with metastatic cancer, 4 with nontuberculous MTB infection, and 24 with reactive hyperplasia. Cervical LN TB was most commonly characterized by multiple enlargement of unilateral cervical lymph nodes, followed by multiple enlargement of bilateral cervical lymph nodes and single enlargement of unilateral cervical lymph nodes (Table 1).

**Table 1:** Main clinical presentation of 612 patients with suspected of cervical LN TB

Location	Number
Left	219
Right	204
Bilateral	189
Main symptoms	
Cervical mass	177
Cervical tenderness	73
Skin redness and swelling	103
Cutaneous sinus	29

Of the 532 cases of cervical LN TB, 403 cases were Xpert MTB/RIF positive, 428 cases were HPE positive, and the sensitivity, specificity, positive predictive value (PPV), and negative predictive value (NPV), were shown in Table 2. Additionally, 476 patients with cervical LN TB were diagnosed with positive CNB with at least one positive result (HPE or Xpert MTB/RIF). Cervical LN TB was diagnosed based on a positive HPE or Xpert MTB/RIF assay result after CNB or both. Statistical analysis showed that compared with Xpert MTB/RIF assay, HPE had higher sensitivity, while Xpert MTB/RIF assay had higher specificity, higher PPV, and lower NPV, and the difference was statistically significant. There were 392 cases of microbial culture in pus, 159 of which were positive(40.5%).

**Table 2:** Comparison of sensitivity, specificity, PPV and NPV of the histopathology examination and Xpert MTB/RIF assay

Test	CRS		Sensitivity (%)	Specificity (%)	PPV (%)	NPV (%)
HPE	428	7	80.4(76.8–83.7)	91.2(82.8–96.4)	98.3(96.7–99.3)	41.2(33.9–48.8)
	104	73				
Xpert MTB/RIF	403	1	75.7(71.9–79.3)	98.7(93.2–99.9)	99.7(98.6–99.9)	37.9(31.3–44.9)
	129	79				

CNB = core needle biopsy; HPE = histopathology examination; MTB = Mycobacterium tuberculosis bacilli; NPV = negative predictive value

PPV = positive predictive value; RIF = rifampicin

Results of complications in perioperative patients with suspected cervical LN TB receiving CNB are as follows: median blood loss (2 ml), duration of operation (11 min), and length of hospital stay (2 days), respectively. No postoperative complications, such as severe bleeding, were noted, and the Clavien–Dindo grading was 2 (Table 3).

**Table 3:** Perioperative outcomes of 102 patients with CNB

Median blood loss, ml (IQR)	1.2(1–2)
Median operative time, min (IQR)	6.7(5–11)
Median length of hospital stay, days (IQR)	1.3(1–2)
7-day postoperative complications, n (%) Overall	102.0(16.6%)
Clavien–Dindo = 1	95.0(15.5%)
Clavien–Dindo = 2	7.0(1.1%)
Clavien–Dindo > 2	0

CNB = core needle biopsy; IQR = interquartile range

Perioperative outcomes: blood loss, operative time, length of hospital stay, 7-day postoperative complications; Postoperative complications: Intraoperative and postoperative bleeding, postoperative pain, and Peripheral lymph node abscess

## Discussion

The gold standard for diagnosing cervical LN TB is pathological diagnosis and microbial culture [8]. The sensitivity of MTB culture for TB diagnosis using surgically excised tissues as specimens has been reported to be 18–93% [9]. Extrapulmonary tuberculosis is an oligobacterial disease, and low bacterial content in specimens often results in reduced sensitivity of MTB culture. In this study, US-CNB was used to diagnose cervical LN TB. US-CNB is currently recognized as a method for obtaining definitive histopathological diagnosis under nonsurgical conditions. The conventional US-CNB procedure is relatively blind and random, particularly in the case of lymph node diseases with a wide range of liquefaction necrosis, highlighting the limitations of this technique.

Scientists have conducted in-depth studies on the application of CEUS to detect lymph nodes [10, 11]. Because cervical LN TB often presents with necrosis, peripheral tissue edema, peripheral abscess, and sinus tract formation [12], CEUS can accurately display blood perfusion in lymph nodes, necrotic areas, and peripheral blood supply. A study has confirmed that lymph node CNB after CEUS can more effectively than CNB obtain diseased lymph node tissue [13]. CEUS can clearly and accurately display the structure of cervical lymph nodes and peripheral blood vessels, allowing for more effective avoidance of vascular injury along the puncture path, thus greatly reducing the occurrence of complications. Therefore, CEUS-assisted US-CNB for cervical LN TB is more scientific.

In this study, tissue specimens were collected by CEUS-assisted US-CNB and analyzed by HPE staining and Xpert MTB/RIF assay. The diagnosis of tuberculosis by HPE is based on the detection of AFB and granulomatous inflammation [14]. Lewinsohn [15] reported a sensitivity of 69–97% in the detection of TB by HPE. Fontanilla [9] reported a sensitivity of 59–88% when HPE was used to detect LN TB. In the present study, the sensitivity of HPE was 80.4%, consistent with that reported in previous studies. In this study, we also used the Xpert MTB/RIF assay to analyze biopsy tissue samples to diagnose LN TB. This assay targets the RNA polymerase β subunit gene (rpoB) of MTB. More than 95% of rifampicin-resistant strains exhibit variation in rpoB [16]. The Xpert MTB/RIF assay has been shown to be effective in the diagnosis of extrapulmonary tuberculosis [17]. Kohli reported that the combined sensitivity and specificity of the Xpert MTB/RIF assay based on the CRS were 42.3% (32.1–52.8%) and 99.8% (99.3–100.0%), respectively, in cerebrospinal fluid samples and 18.9% (11.5–27.9%) and 99.3% (98.1–99.8%), respectively, in pleural fluid samples [14]. The Xpert MTB/RIF assay also has a strong diagnostic value for reproductive system tuberculosis, with a sensitivity and specificity of 62.16% and 100%, respectively [18]. In our study, the sensitivity of the Xpert MTB/RIF assay was slightly higher than that determined in the aforementioned study of cerebrospinal fluid, pleural effusion and genital tuberculosis tissue samples. This may be attributed to differences in the CRS used in our study and the tissue specimens obtained from different organs.

The maximum incubation period for MTB culture is 8 weeks [19], while Xpert MTB/RIF assay can be completed within 2 h only, making it useful for the early diagnosis of cervical LN TB. In addition to detecting TB, the Xpert MTB/RIF assay can also determine whether the TB is resistant to rifampicin [20, 21], which is particularly important for patients with recurrent or treatment-resistant cervical LN TB to determine whether it is a drug-resistant tuberculosis infection, as this directly affects prognosis.

According to our statistical analysis, HPE showed higher sensitivity in the diagnosis of cervical LN TB than the Xpert MTB/RIF assay. Therefore, HPE appears to be more effective in the diagnosis of cervical LN TB when examining tissue specimens. This may be due to the fact that the hospital where the study was conducted is a provincial diagnosis and treatment center for tuberculosis, and the pathologists have extensive experience in the diagnosis of tuberculosis. The lower sensitivity of the Xpert MTB/RIF assay in this study may be attributed to the small amount of MTB present in the specimens or the interference of impurities after a series of complex procedures, such as grinding. Upon submission of specimens for analysis, we included the patients’ clinical symptoms and laboratory examination results on the pathology application form, thus affecting the pathologist’s diagnosis, which could explain why HPE was more sensitive than the Xpert MTB/RIF assay. In this study, the Xpert MTB/RIF assay displayed a high level of specificity. We believe that patients with positive results from the Xpert MTB/RIF assay can serve as a basis for reassessing the clinical diagnosis of LN TB in cases where there is uncertainty about the pathological diagnosis.

We believe that CNB should not target lymph nodes with a high degree of necrosis, and it instead should aim for lymph nodes with a high proportion of enhancement CEUS should be selected as the target lymph nodes. While the diagnostic procedure of cervical LN TB via CNB may result in adverse events such as intraoperative and postoperative bleeding, pain, and peripheral lymph node abscess, our findings indicate that postoperative complications are manageable and generally fall within Clavien–Dindo grade ≦ 2. Therefore, CEUS-assisted US-CNB has strong diagnostic value in the diagnosis of cervical LN TB. This study has some limitations. Firstly, it is a retrospective, single-center study, and the findings of this study should be further validated through multi-center research. Secondly, the success rate of sampling and the incidence of postoperative complications with CEUS-assisted US-CNB are dependent on the experience of the operating physician. Finally, our hospital is a regional referral center, and due to the guidance of medical policy, many patients suspected of having LN TB are referred to our hospital. Our research results have significant implications for further treatment.

## Conclusion

US-CNB assisted by CEUS is a sampling tool for the diagnosis of cervical LN TB, which has strong diagnostic effect and minor complications. HPE and Xpert MTB/RIF assay are recommended for tissue samples after biopsy, because HPE has a high diagnostic efficacy, and patients who test positive for Xpert MTB/RIF can not only promote early diagnosis, but also help to revise the clinical diagnosis of patients with unclear HPE.

## Data Availability

No datasets were generated or analysed during the current study.

## References

[1] WHO (2023) Global tuberculosis Report 2023. World Health Organization

[2] Kang W, Liu S, DU J et al (2022) Epidemiology of concurrent extrapulmonary tuberculosis in inpatients with extrapulmonary tuberculosis lesions in China: a large-scale observational multi-centre investigation. Int J Infect Dis 115:79–85. 10.1016/j.ijid.2021.11.019 34781005 10.1016/j.ijid.2021.11.019

[3] Kang W, Yu J, Du J et al (2020) The epidemiology of extrapulmonary tuberculosis in China: A large-scale multi-center observational study. PLoS One, 15(8): e0237753. 10.1371/journal.pone.0237753. eCollection 2020 10.1371/journal.pone.0237753 PMC7446809 32822367

[4] Koch A (2018) Mycobacterium tuberculosis. Trends Microbiol 26(6):555–556. 10.1016/j.tim.2018.02.012Epub 2018 Mar 23 29580884 10.1016/j.tim.2018.02.012

[5] Kohli M, Schiller I, Dendukuri N, Yao M, Dheda K, Denkinger CM, Schumacher SG, Steingart KR (2021) Xpert MTB/RIF Ultra and Xpert MTB/RIF assays for extrapulmonary tuberculosis and rifampicin resistance in adults. Cochrane Database Syst Rev 1(1):CD012768. 10.1002/14651858.CD012768.pub3 33448348 10.1002/14651858.CD012768.pub3 PMC8078545

[6] Borges WM, Bechara GR, de Miranda MML, de Figueiredo GB, Venturini BA, Laghi CR (2019) Epididymis Tuberculosis: Case report and brief review of the literature. Urol Case Rep 26:100969. 10.1016/j.eucr.2019.100969eCollection 2019 Sep 31367526 10.1016/j.eucr.2019.100969 PMC6656682

[7] Clavien PA, Barkun J, de Oliveira ML, Vauthey JN, Dindo D, Schulick RD, de Santibañes E, Pekolj J, Slankamenac K, Bassi C, Graf R, Vonlanthen R, Padbury R, Cameron JL, Makuuchi M (2009) The Clavien-Dindo classification of surgical complications: five-year experience. Ann Surg 250(2):187–196. 10.1097/SLA.0b013e3181b13ca2 19638912 10.1097/SLA.0b013e3181b13ca2

[8] Machado D, Couto I, Viveiros M (2019) Advances in the molecular diagnosis of tuberculosis: from probes to genomes. Infect Genet Evol 72:93–112 Epub 2018 Nov 30 30508687 10.1016/j.meegid.2018.11.021

[9] Fontanilla JM, Barnes A, von Reyn (2011) CF.Current diagnosis and management of peripheral tuberculous lymphadenitis. Clin Infect Dis 53(6):555–562. 10.1093/cid/cir454 21865192 10.1093/cid/cir454

[10] Zhang X, Wang L, Feng N et al (2021) Reassessing the value of contrast-enhanced ultrasonography in differential diagnosis of cervical tuberculous lymphadenitis and lymph node metastasis of papillary thyroid carcinoma. Front Oncol. ;11:694449. 10.3389/fonc.2021.694449. eCollection 2021 10.3389/fonc.2021.694449 PMC8551861 34722243

[11] Zhao D, He N, Shao YQ et al (2022) The diagnostic value of contrast-enhanced ultrasound for cervical tuberculous lymphadenitis. Clin Hemorheol Microcirc 81(1):69–79. 10.3233/CH-211355 35001882 10.3233/CH-211355 PMC9108573

[12] Lekhbal A, Chaker K, Halily S et al (2020) Treatment of cervical lymph node tuberculosis: when surgery should be performed? A retrospective cohort study. Ann Med Surg (Lond) 55:159–163. 10.1016/j.amsu.2020.05.006 32489658 10.1016/j.amsu.2020.05.006 PMC7256428

[13] Zhao D, Shao Y-Q, Hu J et al (2021) Role of contrast-enhanced ultrasound guidance in core-needle biopsy for diagnosis of cervical tuberculous lymphadenitis. Clin Hemorheol Microcirc 77(4):381–389. 10.3233/CH-201038 33337357 10.3233/CH-201038

[14] Kohli M, Schiller I, Dendukuri N, Dheda K, Denkinger CM, Schumacher SG, Steingart KR (2018) Xpert^®^ MTB/RIF assay for extrapulmonary tuberculosis and rifampicin resistance. Cochrane Database Syst Rev 8(8):CD012768. 10.1002/14651858.CD012768.pub2 30148542 10.1002/14651858.CD012768.pub2 PMC6513199

[15] Lewinsohn DM, Leonard MK, LoBue PA, Cohn DL, Daley CL, Desmond E, Keane J, Lewinsohn DA, Loeffler AM, Mazurek GH, O’Brien RJ, Pai M, Richeldi L, Salfinger M, Shinnick TM, Sterling TR, Warshauer DM, Woods GL (2017) /Centers for Disease Control and Prevention Clinical Practice Guidelines: diagnosis of tuberculosis in adults and children. Clin Infect Dis 64(2):e1–e33. 10.1093/cid/ciw694Epub 2016 Dec 8 Official American Thoracic Society/Infectious Diseases Society of America 27932390 10.1093/cid/ciw694

[16] Thirumurugan R, Kathirvel M, Vallayyachari K, Surendar K, Samrot AV, Muthaiah M (2015) Molecular analysis of rpoB gene mutations in rifampicin resistant Mycobacterium tuberculosis isolates by multiple allele specific polymerase chain reaction in Puducherry, South India. J Infect Public Health 8(6):619–625. 10.1016/j.jiph.2015.05.003 26117709 10.1016/j.jiph.2015.05.003

[17] Bankar S, Set R, Sharma D, Shah D, Shastri J (2018) Diagnostic accuracy of Xpert MTB/RIF assay in extrapulmonary tuberculosis. Indian J Med Microbiol 36(3):357–363. 10.4103/ijmm.IJMM_18_173 30429387 10.4103/ijmm.IJMM_18_173

[18] Pengju Liu HG, Liu Y et al (2022) Application of core needle biopsy in the diagnosis of epididymal tuberculosis: a retrospective analysis of 41 cases. Int J Infect Dis 122:33–37. 10.1016/j.ijid.2022.05.044Epub 2022 May 20 35605951 10.1016/j.ijid.2022.05.044

[19] Wilkinson RJ, Rohlwink UK, Misra UK et al (2017) Tuberculous meningitis. Nat Rev Neurol 13:581–598. 10.1038/nrneurol.2017.120Epub 2017 Sep 8 28884751 10.1038/nrneurol.2017.120

[20] Steingart KR, Schiller I, Horne DJ, Pai M, Boehme CC, Dendukuri N (2014) Xpert^®^ MTB/RIF assay for pulmonary tuberculosis and rifampicin resistance in adults.Cochrane. Database Syst Rev 2014(1):CD009593. 10.1002/14651858.CD009593.pub3 10.1002/14651858.CD009593.pub3 PMC4470349 24448973

[21] Boehme CC, Nabeta P, Hillemann D, Nicol MP, Shenai S, Krapp F, Allen J, Tahirli R, Blakemore R, Rustomjee R, Milovic A, Jones M, O’Brien SM, Persing DH, Ruesch-Gerdes S, Gotuzzo E, Rodrigues C, Alland D, Perkins MD (2010) Rapid molecular detection of tuberculosis and rifampin resistance.N Engl. J Med 363(11):1005–1015. 10.1056/NEJMoa0907847Epub 2010 Sep 1 10.1056/NEJMoa0907847 PMC2947799 20825313

